# Serum Amyloid A Is a Marker for Pulmonary Involvement in Systemic Sclerosis

**DOI:** 10.1371/journal.pone.0110820

**Published:** 2015-01-28

**Authors:** Katja Lakota, Mary Carns, Sofia Podlusky, Katjusa Mrak-Poljsak, Monique Hinchcliff, Jungwha Lee, Matija Tomsic, Snezna Sodin-Semrl, John Varga

**Affiliations:** 1 Department of Rheumatology, University Medical Centre Ljubljana, Ljubljana, Slovenia; 2 Division of Rheumatology, Feinberg School of Medicine, Northwestern University, Chicago, United States of America; 3 Department of Preventive Medicine, Feinberg School of Medicine, Northwestern University, Chicago, United States of America; 4 University of Primorska, Faculty of Mathematics, Natural Sciences and Information Technology, Koper, Slovenia; University of Texas Health Science Center at Houston, UNITED STATES

## Abstract

Inflammation in systemic sclerosis (SSc) is a prominent, but incompletely characterized feature in early stages of the disease. The goal of these studies was to determine the circulating levels, clinical correlates and biological effects of the acute phase protein serum amyloid A (SAA), a marker of inflammation, in patients with SSc. Circulating levels of SAA were determined by multiplex assays in serum from 129 SSc patients and 98 healthy controls. Correlations between SAA levels and clinical and laboratory features of disease were analyzed. The effects of SAA on human pulmonary fibroblasts were studied ex vivo. Elevated levels of SAA were found in 25% of SSc patients, with the highest levels in those with early-stage disease and diffuse cutaneous involvement. Significant negative correlations of SAA were found with forced vital capacity and diffusion capacity for carbon monoxide. Patients with elevated SAA had greater dyspnea and more frequent interstitial lung disease, and had worse scores on patient-reported outcome measures. Incubation with recombinant SAA induced dose-dependent stimulation of IL-6 and IL-8 in normal lung fibroblasts in culture. Serum levels of the inflammatory marker SAA are elevated in patients with early diffuse cutaneous SSc, and correlate with pulmonary involvement. In lung fibroblasts, SAA acts as a direct stimulus for increased cytokine production. These findings suggest that systemic inflammation in SSc may be linked to lung involvement and SAA could serve as a potential biomarker for this complication.

## Introduction

Systemic sclerosis (SSc) is a chronic multisystem disease associated with immune dysregulation, vascular injury and fibrosis [1]. Progressive fibrosis in the skin and lungs are prominent, and ultimately leads to organ failure accounting for the substantial mortality of SSc [2]. Inflammatory infiltrates are observed in a variety of affected organs in early-stage disease [3–5] and are accompanied by elevated circulating levels of inflammatory cytokines and chemokines [6,7]. Serum amyloid A (SAA) is an evolutionarily conserved ˜ 12 kDa acute phase protein [8]. Circulating levels of SAA increase >1000-fold during inflammatory responses in a manner analogous to that of CRP [9]. There are four human isotypes of SAA. Systemic SAA1 and SAA2 are induced in the liver upon stimulation by interleukin-6 (IL-6), interleukin-1β (IL-1β) and tumor necrosis factor-α (TNF-α) [10]. Moreover, SAA can also be produced by macrophages and other extrahepatic cells [11–13] as well as in the lung [14].

The biology of SAA has been investigated extensively [8,15]. SAA is a key mediator in innate immune responses [16], stimulation of cytokines [17], and matrix metalloproteinases [18]. Biological activities include regulation of cholesterol metabolism, insulin resistance and glycemic control [19]. During acute phase, SAA displaces 80% of ApoAI (an apolipoprotein with proven antifibrotic activity [20,21]) from HDL [22]. Elevated levels of SAA are down-regulated by therapy with PPARγ agonist agents [19,23], whereas glucocorticoid treatment did not downregulate extrahepatic expression of SAA [11,24,25]. In chronic inflammatory diseases, such as rheumatoid arthritis, metabolic syndrome or atherosclerosis, prolonged elevation of SAA may contribute to tissue damage and degradation [26–28]. Elevated SAA contributes to AA amyloidosis if abnormal cleavage and deposition occurs in genetically predisposed individuals [29].

SAA was shown to regulate expression of TGF-β, the master regulator of connective tissue remodeling and fibrogenesis [30]. In mice, SAA adenoviral transfer leads to increased plasma TGF-β, and increased biglycan expression. Interestingly, it has been reported that the SAA receptor FPRL-1/FPR2 was involved in TGF-β, as well as in biglycan expression [30]. These studies implicate SAA in extracellular matrix remodeling.

The role of inflammation in SSc, and biomarkers for identifying inflammation, have received scant attention to date. Previous studies showed that erythrocyte sedimentation rate (ESR) is elevated in SSc and predicts mortality [31,32]. ESR is one of the parameters comprising the modified Medsger SSc Disease Severity Scale [33,34]. Levels of CRP are also elevated in SSc, correlate with disease activity and pulmonary function [7,35], and predict pulmonary decline and survival [35]. In contrast to CRP and ESR, little is known to date about SAA in SSc or its role in disease pathogenesis. A small pilot study over three decades ago showed elevated SAA levels in 24% of SSc patients; marked elevations predicted poor survival [36]. In the present study, we sought to determine circulating levels of SAA in SSc, and to correlate these levels with clinical features of the disease. Our findings indicate that SAA levels are elevated in a subset of SSc patients, and correlate with pulmonary involvement and patient-reported outcomes, in particular symptoms related to respiratory dysfunction. *In vitro*, recombinant SAA induced enhanced IL-6 and IL-8 production in fibroblasts explanted from normal human lungs. These findings provide evidence for the occurrence of a systemic inflammatory process in SSc, and suggest a potential for SAA as a biomarker in evaluating patients with SSc.

## Methods

### Patients

One hundred twenty nine consecutive adult patients with SSc, evaluated at the Northwestern Scleroderma Program between February 2009 and April 2010 were included in the study. The study was approved by Northwestern University Institutional Review Board. All patients met the ACR criteria [37]. Serum samples were obtained at scheduled visits after patients signed informed consent approved by Northwestern University Institutional Review Board. Serum was also collected from 98 healthy Caucasian volunteers (65% male, 35% female; median age 43.3 yrs), and processed in a manner identical to that for SSc serum. Samples were stored at −80°C until assayed. Clinical information obtained on the SSc patients at the time of serum collection included demographics, body mass index (BMI), disease duration (defined as interval between first non-Raynaud event and blood sampling as early (up to 36 months) and late (above 36 months)), and modified Rodnan skin score (mRSS, range 0–51). Two-dimensional echocardiography with tissue Doppler, pulmonary function tests (PFT) and high-resolution computed tomography of the chest (HRCT) were performed as clinically indicated. Pulmonary arterial hypertension was diagnosed if the estimated pulmonary artery systolic pressure was ≥ 40 mm Hg on echocardiography or with mean pulmonary artery pressure ≥ 25mm Hg and pulmonary capillary wedge pressure ≤15mmHg on right heart catheterization [38]. Antinuclear antibodies in the serum were detected by indirect immunofluorescence, and antibodies against topoisomerase-1, centromere and RNA polymerase III by latex immunoassay, immunofluorescence and ELISA, respectively (S1 Dataset).

### Determination of serum SAA levels

Serum SAA levels were determined using Milliplex Cardiovascular Disease Panel 2 multiplex assay kits (Millipore, Billerica, MA), according to manufacturer’s instructions. Briefly, samples (1:500 or 1:2000 dilution) and standards, along with sonicated beads were added to the wells. After incubation and washing, antibodies followed by streptavidin-phycoerythrin were added. Wells were then washed and fluorescence measured on a Luminex 100 platform (Luminex, Austin, TX). Two control samples with known concentrations of SAA were included in each analysis. Results were calculated from six standard samples, ranging from 0.08–250 ng/ml in concentration, with four parameter curve fit. The average coefficient of variation from replicates in all analyses was 9%.

### Patient-reported outcomes

During scheduled clinic visits, patients completed six patient-reported outcome questionnaire previously validated in SSc: Short Form 36 (SF-36), Patient Reported Outcomes Measurement Information System (PROMIS-29), Scleroderma Health Assessment Questionnaire-Disability Index (sHAQ-DI)) or Dyspnea Score (Medical Research Council (MRC-DS), St. George’s Respiratory Questionnaire (SGRQ) and Functional Assessment of Chronic Illness Therapy (FACIT) [39–41] (S1 Dataset). Patient-reported outcome measures were provided by 31 patients at a mean of 114 ± 74 days from the time of serum collection.

### Effects of SAA on lung fibroblast in vitro

Fibroblasts explanted from healthy adult lungs (Lonza, Walkersville, MD, USA) were used. Fibroblasts were seeded in 6-well plates and maintained in fibroblast basal medium with growth supplements (Lonza, Walkersville, MD, USA) and 20% FBS in a humidified atmosphere of 5% CO2 at 37°C. Sub-confluent low passage fibroblasts were incubated with human recombinant SAA (Peprotech EC Ltd, London, UK) at indicated concentrations for 24h. Levels of IL-6 were determined in culture supernatants by ELISA (Invitrogen, Carlsbad, CA, USA) following the manufacturer’s instructions. RNA was isolated from confluent fibroblasts using RNeasy Plus Micro Kits (Qiagen, Hilden, DE) and reverse transcribed using Reverse transcription System (Promega, Madison, WI, USA). StellARray platforms were used to measure gene expression (Bar Harbor BioTechnology, Trenton, ME, USA).

### Statistical analysis

The normality of distribution of SAA levels was determined by Kolmogorov-Smirnov test. Due to non-normal distribution of the data, summary statistics are expressed as medians and interquartile ranges and nonparametric tests were performed. Mann-Whitney U tests or Kruskal-Wallis tests were used to compare SAA levels. Cut-off was defined as 95 percentile of healthy controls. Spearman’s rank correlations were calculated to measure the correlation between SAA levels and various clinical/laboratory parameters, which accommodated skewedness in measures of SAA. Because SAA was found not to correlate with age, gender or ethnicity, partial correlation was not used for adjustment. Odds ratios of increased SAA were calculated with 95% confidence interval (CI). Data were analyzed using SPSS Statistics 17 (Chicago, IL). P < 0.05 was considered statistically significant.

## Results

### SAA levels are elevated in SSc

**Figure 1:**
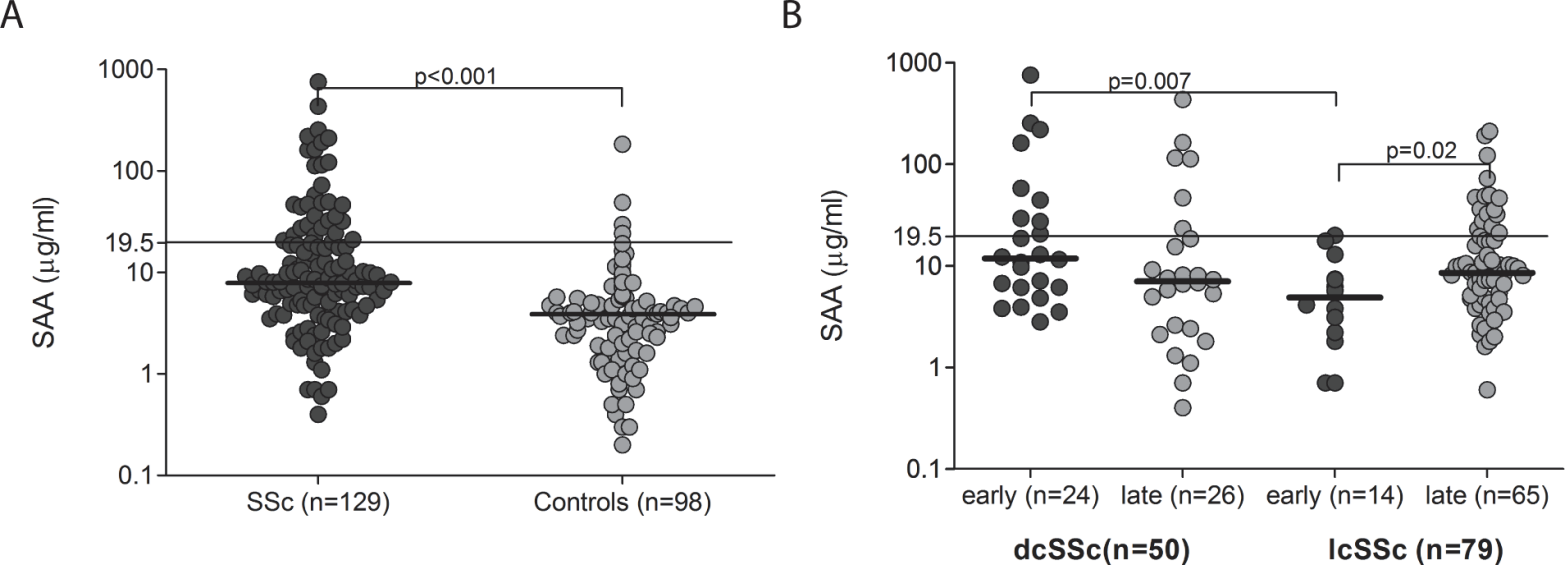
Levels of circulating SAA are elevated in SSc. SAA levels were determined in SSc patients with early (<36 months) and late (>36 months) stage disease and healthy controls. The horizontal line signifies the cut-off value (19.5 μg/ml). Bold horizontal lines represent the medians.

Levels of SAA were significantly higher in patients with SSc compared to healthy controls (U = 3419, p<0.000) (Fig. 1A). Gender, ethnicity, age or clinical SSc subtype (dcSSc or lcSSc) did not significantly influence levels of SAA. Using a cut-off value of 19.5 μg/ml determined in 98 healthy controls, 37% patients with early dcSSc, but none of patients with early lcSSc, were found to have elevated SAA levels (U = 80, p = 0.007). Early stage dcSSc (defined as disease duration < 36 month from the first non-Raynaud symptom of SSc) was associated with higher SAA levels compared with late-stage disease (U = 222, p = 0.08), whereas an opposite trend was seen in patients with lcSSc (U = 269, p = 0.02) (Fig. 1B). No significant differences in SAA levels were detected when patients were classified based on their scleroderma specific autoantibody profiles.

### SAA levels are associated with patient reported outcome measures

Respiratory symptoms were measured by SGRQ and by FACIT. Both the SGRQ symptoms (frequency of respiratory symptoms over a preceeding period (U = 40, p = 0.04)), as well as dyspnea severity and dyspnea related functional limitation scores on the FACIT (U = 31, p = 0.03 and U = 26, p<0.01, respectively) were significantly worse in patients with elevated SAA (Table 1). Moreover, patients with elevated SAA levels had significantly different scores on SF-36, PROMIS-29 and HAQ-DI physical and emotional components (S1 Table).

**Table 1:** Correlation of circulating SAA with respiratory symptoms.

	SAA<19.5 μg/ml			SAA>19.5 μg/ml			Significance Mann Whitney U;p
	n	median	IQR	n	median	IQR	
SGRQ symptoms	23	8	4–31	7	31	21–56	40;p = 0.044
SGRQ activity	22	41	0–66	7	35	23–92	56;p = 0.290
SGRQ impact	21	3	0–12	7	10	0–54	51;p = 0.210
SGRQ total	21	17	1–28	7	20	8–66	45;p = 0.129
FACIT dyspnea	24	40	33–45	6	46	42–61	31;p = 0.033
FACIT functional limitation score	23	35	29–46	7	50	43–64	26;p = 0.007
MRC-DS	23	1	0–1	6	0.5	0–1.5	66;p = 0.880

SGRQ, St. George’s respiratory questionnaire; FACIT, Functional assessment of chronic illness therapy; MRC-DS, Medical Research Council dyspnea score. PRO, Patient-reported outcome. PRO measures were collected within 6 months of serum collection.

### Elevated serum SAA levels are associated with pulmonary complications

**Figure 2:**
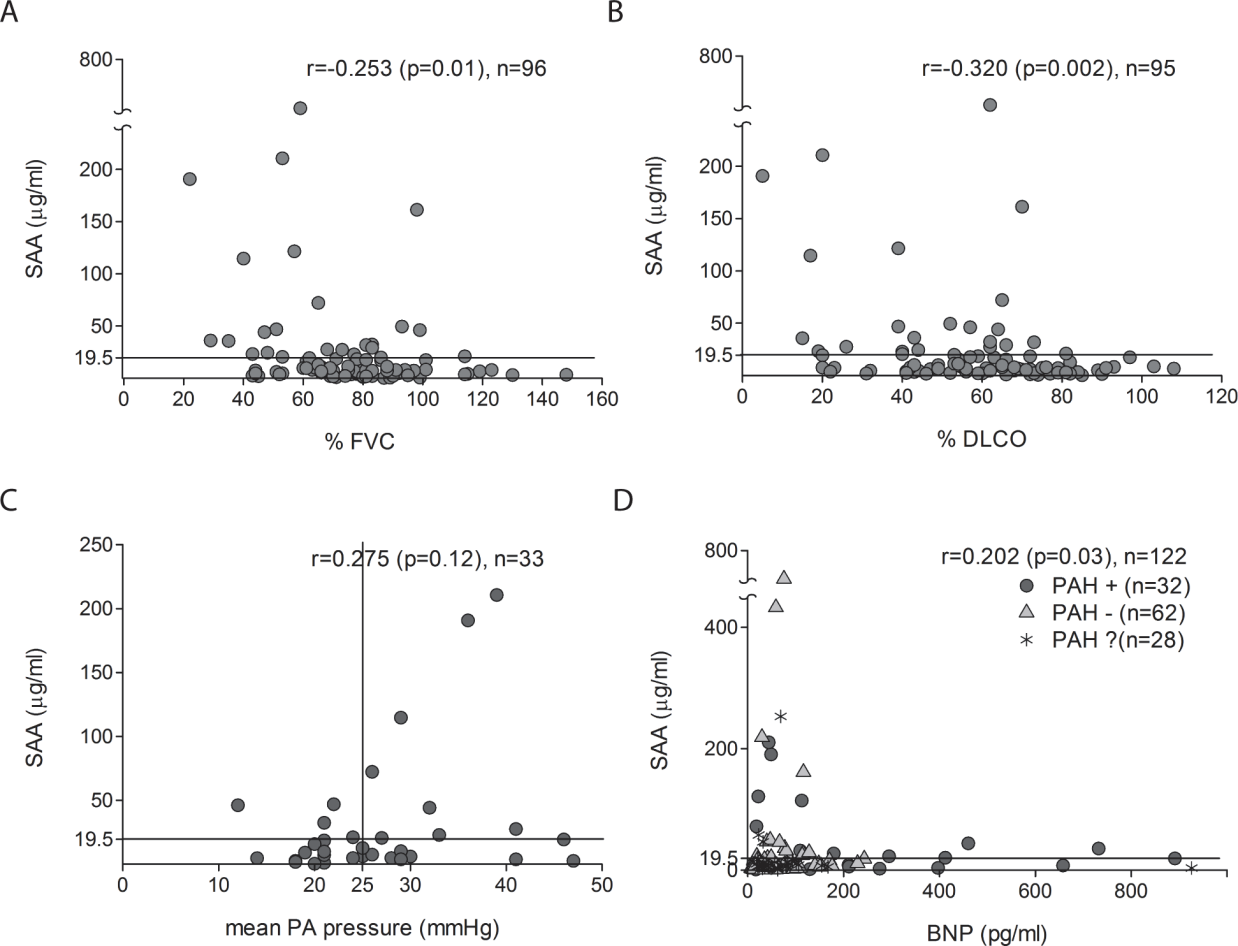
SAA levels are correlated with pulmonary function. Correlation between serum levels of SAA and pulmonary function tests (A, B); pulmonary artery pressure (C); serum BNP levels (D). The horizontal line represents the SAA cut-off (19.5μg/ml) and vertical line cut-off for pulmonary arterial hypertension (right heart catheterization mPAO≥25 mmHg). Spearman correlation coefficient (r), p value, and number of patients (n) are shown.

Patients with elevated SAA had significantly impaired pulmonary function (Table 2). In particular, SAA levels were inversely correlated with FVC (r = −0.253, p = 0.01) and DLCO (r = −0.320, p = 0.002) (Fig. 2A and 2B). Moreover, different radiologic patterns of lung involvement were associated with significant differences (Kruskal-Wallis χ^2^_df = 3_ = 9.23, p = 0.03) in SAA levels (S2 Table). Of note, patients with elevated SAA were 6.3 times more likely to have reduced DLCO (< 70% of predicted; 95% CI 1.36–28.91), and 3.7 times more likely to show a honeycomb or reticulation pattern on chest HRCT (95% CI 1.34–10.17) (S3 Table). In addition to interstitial lung disease, a correlation between SAA levels and mean pulmonary artery pressure was also noted (r = 0.275, n = 33, p = 0.12) (Fig. 2C). This correlation became even stronger (r = 0.702, n = 11, p = 0.02) in Scl-70 positive patients (r = 0.721, n = 11, p = 0.01). Moreover, serum SAA levels positively correlated with serum BNP levels (r = 0.202, n = 122, p = 0.03) (Fig. 2D).

**Table 2:** Comparison of clinical and laboratory features of the SSc patients with normal and elevated SAA

Parameter	SAA<19.5 μg/ml			SAA>19.5 μg/ml			Significance Mann Whitney U; P
	n (M/F)	median	IQR	n (M/F)	median	IQR	
SAA (μg/ml)	97 (13/84)	6.6	3.5–9.6	32 (7/25)	46.8	28–151.5	0.00; p<0.001
Age (yrs)	97 (13/84)	53	46.5–60	32 (7/25)	52.5	44.3–84.5	2976; p = 0.20
FVC (% predicted)	71 (6/65)	78	66–88.0	25 (3/22)	65	47.5–84.5	614; p = 0.02
DLCO (% predicted)	70 (6/64)	65	52.8–76.3	25 (3/22)	52	32.5–63	480; p = 0.001
BNP (pg/ml)	93 (13/80)	44.4	24.5–94.3	29 (6/23)	124.5	40.5–115.7	1049; p = 0.07
MRSS (0–51)	92 (12/80)	6	4–13	32 (7/25)	6	4–19	1326; p = 0.40
RVSP (mmHg)	11 (0/11)	35	34–40	8 (1/7)	31	27–39.8	31; p = 0.30
PASP (mmHg)	44 (7/37)	34	28–41.8	13 (1/12)	35	28.5–53	257; p = 0.59
Mean PA (mmHg)	21 (4/17)	24	20.0–29.0	12 (2/10)	28	22.5–35.3	89; p = 0.17
BMI	93 (12/81)	25.9	23–29.5	32 (7/25)	25.8	21.8–29.2	1338; p = 0.40

FVC, forced vital capacity (percent predicted); DLCO, diffusing capacity for carbon monoxide (percent predicted); BNP, brain natriuretic peptide; MRSS, modified Rodnan skin score; RVSP, right ventricular systolic pressure; PASP, pulmonary artery systolic pressure estimated by Echo/Doppler measurement; PA, pulmonary artery pressure determined by right heart catheterization; BMI, body mass index; M/F, male/female. Median and intraquartile range (IQR) are shown due to non-normality of data distribution. Mann-Whitney U tests were used to compare groups with elevated and normal SAA levels for each parameter.

### SAA levels correlate with other markers of inflammation

**Figure 3:**
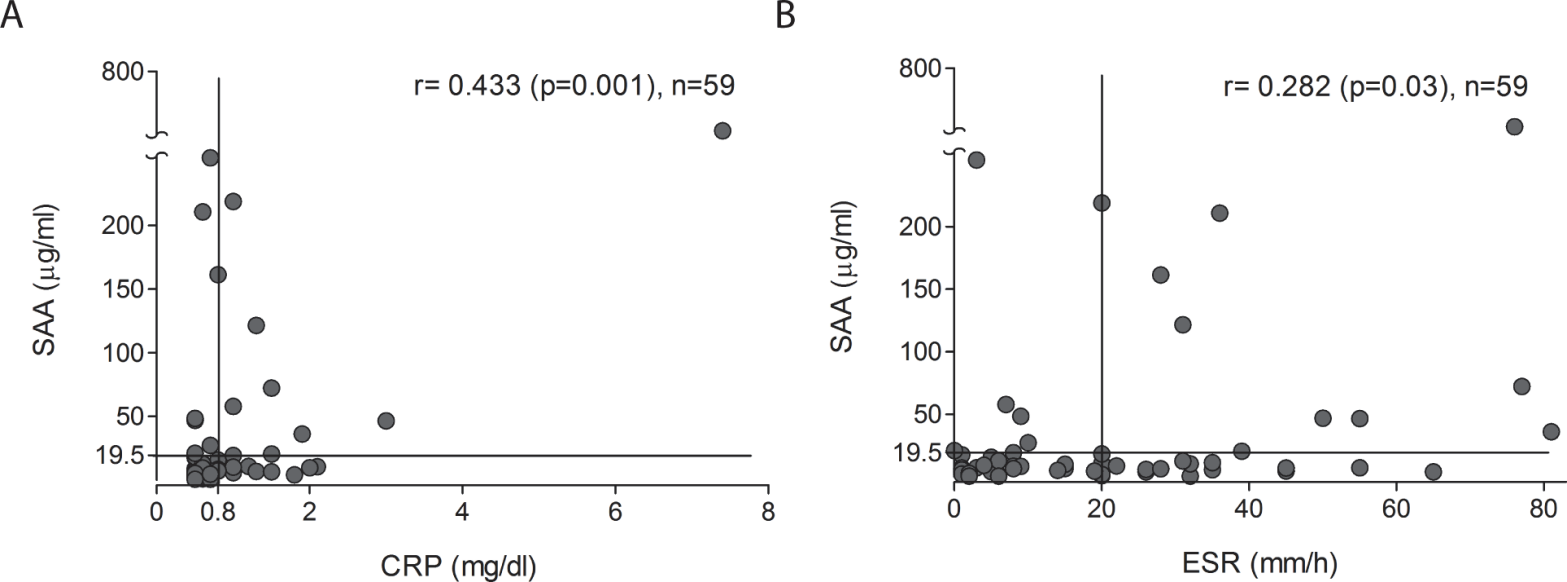
SAA levels correlate weakly with CRP and ESR. Vertical lines indicate cut-off values for CRP (0.8 mg/dl) and ESR (20 mm/h), horizontal line shows cut-off for SAA (19.5 μg/ml). Spearman correlation coefficient (r), p value and number of patients (n) are shown.

The ESR and serum levels of CRP were elevated in 37.3% and 28.8% of patients, respectively. Both inflammation markers correlated with SAA (SAA/CRP r = 0.433, p = 0.001; SAA/ESR r = 0.282, p = 0.030) (Table 3 and Fig. 3), as well as with FVC and DLCO (Table 3).

**Table 3:** Correlations between serum levels of SAA, CRP, and ESR.

	Measured range	Cut-off value	Numbers of patients above cut-off	**Correlation with FVC** n = 36	**Correlation with DLCO** n = 36
SAA	0–753 μg/ml	19.5 μg/ml	25.4% (15/59)	r = −0.391; p = 0.02	r = −0.294; p = 0.08
CRP	0.5–7.4 mg/dl	0.8 mg/dl	28.8% (17/59)	r = −0.516; p = 0.001	r = −0.358; p = 0.03
ESR	0–81 mm/h	20 mm/h	37.3% (22/59)	r = −0.486; p = 0.001	r = −0.414; p = 0.01

r—Spearman correlation coefficients; p—significance

### SAA increases IL-6 expression in pulmonary fibroblasts

**Figure 4:**
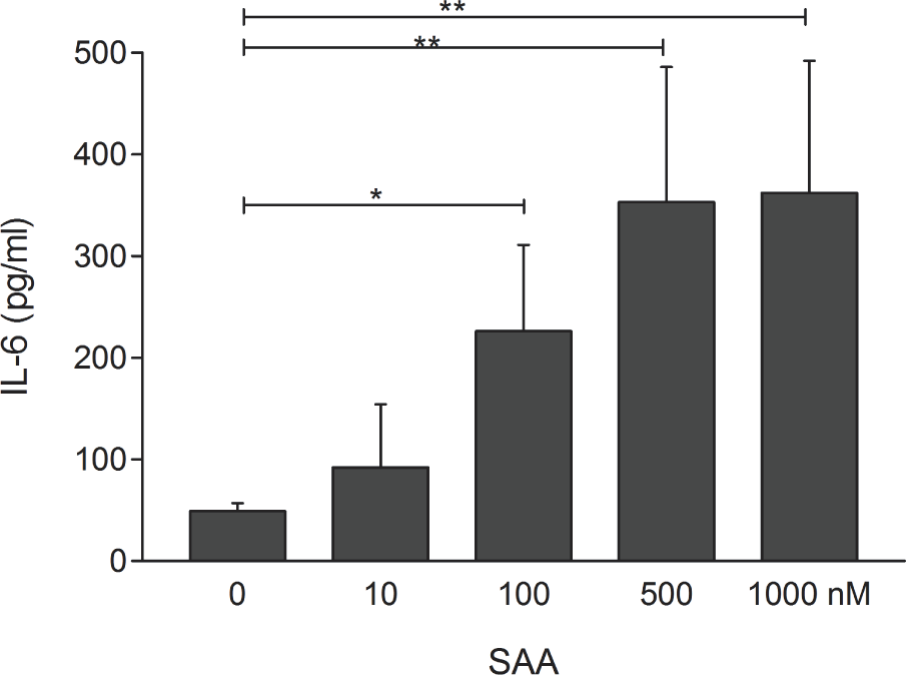
SAA stimulates IL-6 production in lung fibroblasts. Subconfluent human lung fibroblasts in culture were incubated with indicated concentrations of SAA for 24 h. Secreted IL-6 in the culture media was measured by ELISA. Results are means ± standard deviations of triplicate determinations. * p<0.05; ** p<0.01.

To explore the biological activities of SAA on fibroblasts, the primary effector cells of fibrosis linked to the pathogenesis of SSc, low-passage fibroblasts explanted from healthy lungs were incubated with recombinant SAA, followed by determination of secreted IL-6 and changes in fibroblast gene expression in culture. The results indicated that SAA caused a dose-dependent increase in IL-6 secretion (Fig. 4). Moreover, SAA enhanced the expression of IL-6 and IL-8 mRNA (Table 4). In addition, SAA also stimulated the production of matrix metalloproteinases MMP-1 and MMP-12. In contrast, no consistent effect of SAA on collagen gene expression was observed (Table 4).

**Table 4:** Effects of SAA in human lung fibroblasts.

Target gene	Effect of SAA (change in expression [-fold])
Col1a1	0.64 ± 0.20
Col1a2	0.87 ± 0.18
CTGF	1.02 ± 0.72
IL-1β	0.49 ± 0.02
IL-6	8.48 ± 0.65
IL-8	111.27 ± 67.04
MMP-1	2.49 ± 0.65
MMP-12	7.39 ± 0.81
PAI-1	1.97 ± 0.04

Healthy lung fibroblasts in culture were incubated with human recombinant SAA (1 μM) for 24 h. Cultures were harvested, and mRNA levels for selected genes were determined using StellArray assays. Results are expressed as mean—fold change ± standard deviation in SAA-treated compared to control cultures, normalized with GAPDH levels. Experiments were performed in duplicates. Note: dramatic increase in interleukin 6 (IL-6), interleukin 8 (IL-8) and a modest increase in PAI-1, MMP-1 and MMP-12 mRNA levels; in contrast, no significant effect on levels of CTGF, Col1a1 and Col1a2 mRNA. CTGF, connective tissue growth factor; MMP, matrix metalloproteinase; PAI-1, plasminogen activator inhibitor-1.

## Discussion

We show here that circulating levels of the inflammatory marker SAA are elevated in patients with SSc. Elevated SAA levels are associated with signs and symptoms of pulmonary involvement, as well as health-related quality of life measures. In particular, levels of SAA were found to correlate with measures of pulmonary function and radiologic evidence of SSc-associated interstitial lung disease. Furthermore, SAA levels were significantly correlated with PA pressure, in a manner analogous to recent findings in patients with idiopathic pulmonary arterial hypertension [42]. Exposure of healthy lung fibroblasts in culture to SAA resulted in stimulation of the expression of IL-6 and IL-8, two cytokines previously implicated in the pathogenesis of SSc.

The levels of SAA were only modestly correlated with those of the inflammatory markers CRP and ESR. While levels of CRP and ESR were elevated in 29 and 37% of SSc patients, respectively, the correlation with SAA was less than 0.5, revealing unexpected differences in these three inflammatory parameters in SSc. Our observations are broadly consistent with previous studies examining CRP and ESR in SSc [7,35]. Chronic inflammation and fibrosis are often linked, particularly in interstitial lung disease. For instance, in patients with sarcoid lung disease, SAA correlated with collagen deposition and lung fibrosis [12] and negative correlation of lung functions and SAA was found [43].

Recombinant SAA potently stimulated the production of IL-6 and IL-8 in lung fibroblasts in culture. Importantly, these stimulatory effects of SAA on cytokine gene expression occurred at physiologic concentrations of SAA.

We previously reported that SAA stimulated IL-6 in human endothelial cells in culture [44,45] and IL-8, MMP-3 proteins and NF-ƘB DNA binding activity were up-regulated by SAA in fibroblast-like synoviocytes [46]. In this study we report stimulation of IL-6 in lung fibroblasts at the mRNA level and secreted cytokine production. IL-6 is emerging as a potentially important mediator of fibrosis in SSc. In fibroblasts, SAA has been recently shown to trigger a TLR2-dependent innate immune pathway, contributing to induction of IL-6, and potentially linking SAA to innate immunity and fibrosis in SSc [47]. IL-6 is implicated in the regulation of collagen gene expression and extracellular matrix production [48,49]. Furthermore, levels of IL-6 are elevated in serum and lesional tissue of patients with SSc [50,51]. Treatment of SSc patients with anti-IL-6 intervention was shown to have beneficial effects in a small clinical trial [52]. IL-8 is a multifunctional chemokine produced primarily by macrophages, and exerting potent effects on chemotaxis and angiogenesis. Scleroderma fibroblasts spontaneously secrete IL-8 [53]. We and others have shown that levels of IL-8 are elevated in the serum, as well as in bronchoalveolar lavage fluid, from patients with SSc [54–56].

The present results demonstrate elevated circulating SAA levels in a subset of SSc patients that are correlated with symptoms and signs of SSc-associated pulmonary involvement. The biological implications of these findings remain to be elucidated. It is noteworthy, however, that in lung fibroblasts, SAA acts as a direct stimulus for the synthesis of IL-6 and IL-8, mediators implicated in the pathogenesis of SSc and its pulmonary complications. Longitudinal studies to determine if baseline SAA levels in SSc predict disease activity or progression, and whether changes in SAA levels over time correlate with changes in measures of disease activity, seem warranted.

## Supporting Information

S1 Dataset (XLSX)

S1 Table Patient-reported outcomes in patients with normal and elevated SAA. Short form-36 (SF-36), patient-reported outcomes measurement information system (PROMIS-29) and health assessment questionnaire-disability index (sHAQ-DI) were collected within 6 months of serum collection.
(DOCX)

S2 Table SAA levels and chest radiologic patterns. SAA median levels associated with different radiologic ILD patterns on high resolution computerized tomography (HRCT) of the chest. IQR, interquartile range. Kruskal Wallis test to compare SAA levels among different HRCT patterns was significant (p = 0.03), so Mann Whitney pairwise comparisons were performed and adjusted for overall p-value using Bonferroni correction.
(DOCX)

S3 Table SAA levels are associated with radiologic patterns and pulmonary function tests. HRCT, high resolution computerized tomography; FVC, forced vital capacity; DLCO, carbon monoxide diffusing capacity.
(DOCX)
