# Supplementary material for: Serum Amyloid A Is a Marker for Pulmonary Involvement in Systemic Sclerosis
Source: PLoS One. 2015 Jan 28;10(1):e0110820. doi: 10.1371/journal.pone.0110820 (PMC4321755; doi:10.1371/journal.pone.0110820)
Supplement: S1 Table — Short form-36 (SF-36), patient-reported outcomes measurement information system (PROMIS-29) and health assessment questionnaire-disability index (sHAQ-DI) were collected within 6 months of serum collection. (DOCX) [file pone.0110820.s002.docx]

**Table S1:** Patient-reported outcomes in patients with normal and elevated SAA

|  | **SAA<19.5 μg/ml** | | | **SAA>19.5 μg/ml** | | | Significance  Mann Whitney U;p |
| --- | --- | --- | --- | --- | --- | --- | --- |
| **SF-36** | n | median | IQR | n | median | IQR |  |
| Physical functioning | 24 | 67 | 45-97 | 7 | 35 | 10-60 | **39; p=0.03** |
| Role limitations - physical | 24 | 71 | 50-100 | 7 | 25 | 0-62 | **30; p=0.01** |
| Bodily pain | 24 | 74 | 41-84 | 7 | 31 | 22-51 | **31;p=0.01** |
| General health | 24 | 47 | 32-75 | 7 | 40 | 10-47 | 46;p=0.07 |
| Vitality | 24 | 53 | 43-75 | 7 | 37 | 31-50 | **98;p=0.03** |
| Social function | 24 | 75 | 50-100 | 7 | 75 | 25-75 | 62;p=0.30 |
| Role limitation - emotional | 24 | 100 | 54-100 | 6 | 58 | 45-64 | **31;p=0.02** |
| Mental health | 24 | 75 | 56-88 | 6 | 45 | 40-76 | **34;p=0.02** |
| Standardized physical component | 24 | 43 | 32-53 | 6 | 31 | 23-42 | **33;p=0.04** |
| Standardized physical component | 24 | 52 | 40-58 | 6 | 38 | 33-52 | 40;p=0.10 |
| **PROMIS-29** |  |  |  |  |  |  |  |
| T - score fatigue | 24 | 51 | 44-59 | 7 | 60 | 53-64 | 44.5;p=0.06 |
| T - score physical | 24 | 48 | 35-56 | 7 | 36 | 26-47 | **32;p=0.01** |
| T - score depression | 24 | 48 | 41-55 | 7 | 60 | 55-60 | **30;p=0.01** |
| T - score pain | 24 | 51 | 41-62 | 7 | 62 | 58-71 | **35;p=0.02** |
| T - score satisfaction | 24 | 48 | 42-64 | 7 | 39 | 29-43 | **33;p=0.02** |
| T - score sleep | 24 | 52 | 41-55 | 7 | 55 | 49-59 | 54;p=0.15 |
| T - score anxiety | 24 | 48 | 40-55 | 7 | 60 | 57-63 | **26;p=0.01** |
| **sHAQ-DI** | 23 | 0.4 | 0-1.4 | 7 | 1.5 | 0.6-2.1 | **33;p=0.02** |

Short form-36 (SF-36), patient-reported outcomes measurement information system (PROMIS-29) and health assessment questionnaire-disability index (sHAQ-DI) were collected within 6 months of serum collection.
