# Supplementary material for: Serum Amyloid A Is a Marker for Pulmonary Involvement in Systemic Sclerosis
Source: PLoS One. 2015 Jan 28;10(1):e0110820. doi: 10.1371/journal.pone.0110820 (PMC4321755; doi:10.1371/journal.pone.0110820)
Supplement: S2 Table — SAA median levels associated with different radiologic ILD patterns on high resolution computerized tomography (HRCT) of the chest. IQR, interquartile range. Kruskal Wallis test to compare SAA levels among different HRCT patterns was significant (p = 0.03), so Mann Whitney pairwise comparisons were performed and adjusted for overall p-value using Bonferroni correction. (DOCX) [file pone.0110820.s003.docx]

**Table S2:** SAA levels and chest radiologic patterns

| **HRCT pattern** | **n** | **SAA**  **(µg/ml)** | **IQR** | ***P* value** |
| --- | --- | --- | --- | --- |
| No ILD pattern | 30 | 7.7 | 3.8-17.7 | 0.02  0.02  0.02  0.001  0.01 |
| Ground glass | 13 | 4.1 | 2.0-14.0 |  |
| Reticulation or honeycombing | 10 | 29.7 | 9.8-101.9 |  |
| ≥1 ILD pattern | 33 | 8.8 | 3.8-27.4 |  |

SAA median levels associated with different radiologic ILD patterns on high resolution computerized tomography (HRCT) of the chest. IQR, interquartile range. Kruskal Wallis test to compare SAA levels among different HRCT patterns was significant (p=0.03), so Mann Whitney pairwise comparisons were performed and adjusted for overall p-value using Bonferroni correction.
