# Supplementary material for: Serum Amyloid A Is a Marker for Pulmonary Involvement in Systemic Sclerosis
Source: PLoS One. 2015 Jan 28;10(1):e0110820. doi: 10.1371/journal.pone.0110820 (PMC4321755; doi:10.1371/journal.pone.0110820)
Supplement: S3 Table — HRCT, high resolution computerized tomography; FVC, forced vital capacity; DLCO, carbon monoxide diffusing capacity. (DOCX) [file pone.0110820.s004.docx]

**Table S3:** SAA levels are associated with radiologic patterns and pulmonary function tests

|  |  | **SAA** | | **Odds ratio**  **(95% CI)** | | ***P* value** |  |
| --- | --- | --- | --- | --- | --- | --- | --- |
|  |  | **<19.5 µg/ml** | **>19.5 µg/ml** |  |  |  |  |
| **HRCT pattern** |  |  |  |  | |  |  |
| Ground glass | pos | 28 | 13 | **0.78**  (0.31, 1.99) | | 0.61 |  |
|  | neg | 33 | 12 |  |  |  |  |
| Reticulation | pos | 22 | 13 | **1.92**  (0.75, 4.93) | | 0.17 |  |
|  | neg | 39 | 12 |  |  |  |  |
| Honeycombing | pos | 8 | 9 | **3.73**  **(1.24, 11.24)** | | **0.02** |  |
|  | neg | 53 | 16 |  |  |  |  |
| Honeycombing or reticulation | pos | 25 | 18 | | **3.70**  **(1.347, 10.17)** | **0.009** | |
|  | neg | 36 | 7 | |  |  |  |
| **Pulmonary function tests** |  |  |  | |  |  | |
| FVC (% predicted) | >70 | 37 | 10 | | **1.99**  (0.77, 5.17) | 0.16 | |
|  | <70 | 26 | 14 | |  |  |  |
| DLCO (% predicted) | >70 | 24 | 2 | | **6.27**  **(1.36, 28.91)** | **0.02** | |
|  | <70 | 44 | 23 | |  |  |  |

HRCT, high resolution computerized tomography; FVC, forced vital capacity; DLCO, carbon monoxide diffusing capacity.
